# Language Modelling Techniques for Analysing the Impact of Human Genetic Variation

**DOI:** 10.1177/11779322251358314

**Published:** 2025-09-02

**Authors:** Megha Hegde, Jean-Christophe Nebel, Farzana Rahman

**Affiliations:** 1 School of Computer Science and Mathematics, Kingston University, London, UK

**Keywords:** Variant effect prediction, large language models, small language models, evolution of language models, genomics

## Abstract

Interpreting the effects of variants within the human genome and proteome is essential for analysing disease risk, predicting medication response, and developing personalised health interventions. Due to the intrinsic similarities between the structure of natural languages and genetic sequences, natural language processing techniques have demonstrated great applicability in computational variant effect prediction. In particular, the advent of the Transformer has led to significant advancements in the field. However, transformer-based models are not without their limitations, and a number of extensions and alternatives have been developed to improve results and enhance computational efficiency. This systematic review investigates over 50 different language modelling approaches to computational variant effect prediction over the past decade, analysing the main architectures, and identifying key trends and future directions. Benchmarking of the reviewed models remains unachievable at present, primarily due to the lack of shared evaluation frameworks and data sets.

## Introduction

Understanding the impact of genetic variants is crucial for unravelling gene regulation mechanisms and disease causality. As we enter the era of personalised medicine, it has become of great interest to understand how an individual’s genetic makeup can impact their risk of developing a particular disease or their response to a specific treatment or medication.^1,2^

Any change in a coding region can directly affect the function of the associated protein; hence, certain gene mutations can be linked with specific diseases. While Mendelian (monogenic) diseases, such as cystic fibrosis and haemophilia, are caused by mutations in a single gene,^3,4^ polygenic diseases, including many cancers,^5,6^ result from combinations of mutations.^7,8^ Variation in the non-coding region of the genome is more challenging to interpret than that in the coding region, as variants impact disease-related genes by altering processes such as transcription, chromatin folding, or histone modification.^9,10^

Indeed, linguistic metaphors, from alphabets to grammars, have been readily used to describe the molecular world since the discovery of the structure of DNA in the 1950s.^11,12^ For instance, as genetic sequences are comprised of nucleotides or amino acids represented as letters, the sequences themselves can be represented as strings of letters, and processed in a way that is analogous to human language.^13,14^

Although Noam Chomsky formed the basis of modern language modelling in the 1950s,^15^ the field has advanced considerably over the decades. A pivotal point was the development of the transformer in 2017,^16^ which sparked a discernible shift towards the use of so-called large language models (LLMs) to solve a plethora of language modelling tasks in bioinformatics, including variant effect prediction.^17,18^ These LLMs are transformer-based models with billions of parameters, trained on large corpora of sequence data, and have been favoured due to their ability to accurately model long-range dependencies within sequences.^19,20^

Large language models have been used extensively in bioinformatics, and many excellent reviews have been published on several aspects. However, existing review papers either focus broadly on LLMs for general bioinformatics applications^21-23^ or on an overview of machine learning techniques for variant effect prediction.^18^ Our review, however, focuses specifically on the applications of language models for variant effect prediction. In addition, we provide an in-depth analysis of LLMs, including post-transformer techniques, which are as yet underrepresented in the existing reviews. Hence, this review addresses this gap by first presenting an introduction to variant effect prediction and biological language modelling, before entering an in-depth exploration of language models applied to the prediction of effects of genetic variations within DNA, RNA, and protein sequences. Following a brief presentation of the history of language modelling, in line with the rapid advancement in the field, the core of the review covers models produced since the inception of the transformer in 2017. This review focuses on variants within the human genome and their impacts on disease causality; however, models trained on multi-species data are also considered.

## Methods

This review details language modelling approaches to predicting the effects of variants (mutations) in DNA, RNA, and protein sequences. The papers in this review were selected from exhaustive searches across Google Scholar and Science Direct, using the following keywords and phrases: ‘variant effect prediction’, ‘mutation effect prediction’, ‘language modelling’, ‘natural language processing’, and ‘large language model’. Selected papers were required to fulfil the following criteria: (1) to capture recent innovations and emerging trends in this rapidly evolving field, the review was limited to publications from the past decade (2014-2024), along with a select few from early 2025 published prior to the submission of this article, (2) input data must include a DNA, RNA, or protein sequence, (3) the task must be variant effect prediction, and (4) the technique must involve language modelling, for instance, a traditional natural language processing (NLP) approach, a convolutional neural network, or an LLM.

## Background

As this article details the applications of language modelling to variant effect prediction tasks, this section provides a brief introduction to both aspects – variant effect prediction and NLP – to set out the main problems in the field, and the technologies that can be used to address them.

### Variant effect prediction

Uncovering the associations between genetic variants and human diseases necessitates an understanding of the many different possible types of variants. The variants most commonly explored in the field are single base-pair substitutions, referred to as single-nucleotide polymorphisms (SNPs). Still, a small number of models have been developed to analyse the combined effect of several SNPs.^5,24^ While several single base-pair substitutions can co-occur independently, they can also occur as a single event; in such cases, they are referred to as multiple base-pair substitutions.^25^ However, there is no evidence they have been addressed in the literature. In addition to substitutions, 2 other significant forms of variation are insertions and deletions, collectively known as indels. Insertion refers to the case where additional nucleotides are inserted into a genetic sequence, while deletion refers to the case where nucleotides are deleted from such a sequence. Similarly to substitutions, these events can occur across single or multiple nucleotides. While some papers have investigated indel effect prediction,^26-29^ this has been explored to a substantially lesser extent than substitutions.

**Figure 1.**
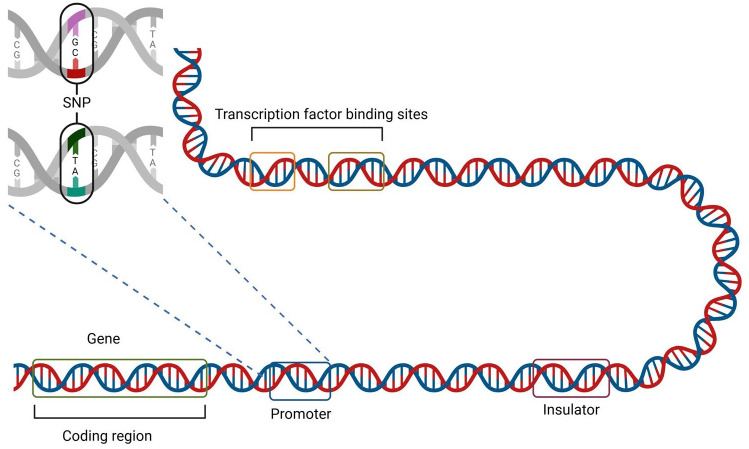
Illustration of coding vs non-coding DNA, and an SNP in a promoter region, for a eukaryotic cell. Non-coding DNA consists of transcription factors, such as promoters, and transcription factor binding sites. Promoters drive the initiation of transcription.^32^ Other cis-regulatory elements (CREs) include enhancers and silencers, which positively and negatively regulate gene expression, respectively. Insulators are an additional type of CRE, which interact with nearby CREs and can block distal enhancers or regulate chromatin interactions.^33^ Source: Created in BioRender. Hegde (2024) https://BioRender.com/e16b233.

Existing work focuses largely on variants within genes, which code for proteins. However, these protein-coding regions comprise less than 2% of the human genome.^30^ As illustrated in Figure 1, variants can also occur in the non-coding regions of the genome, including in regulatory elements such as promoters and enhancers. In fact, 90% of disease-associated variants identified by genome-wide association studies have mapped to non-coding regions, and the majority of these remain unannotated.^31^ Hence, the discovery of non-coding variant effects remains a largely untapped source of potential knowledge that could aid in illuminating human disease mechanisms.

### Natural language processing

**Figure 2.**
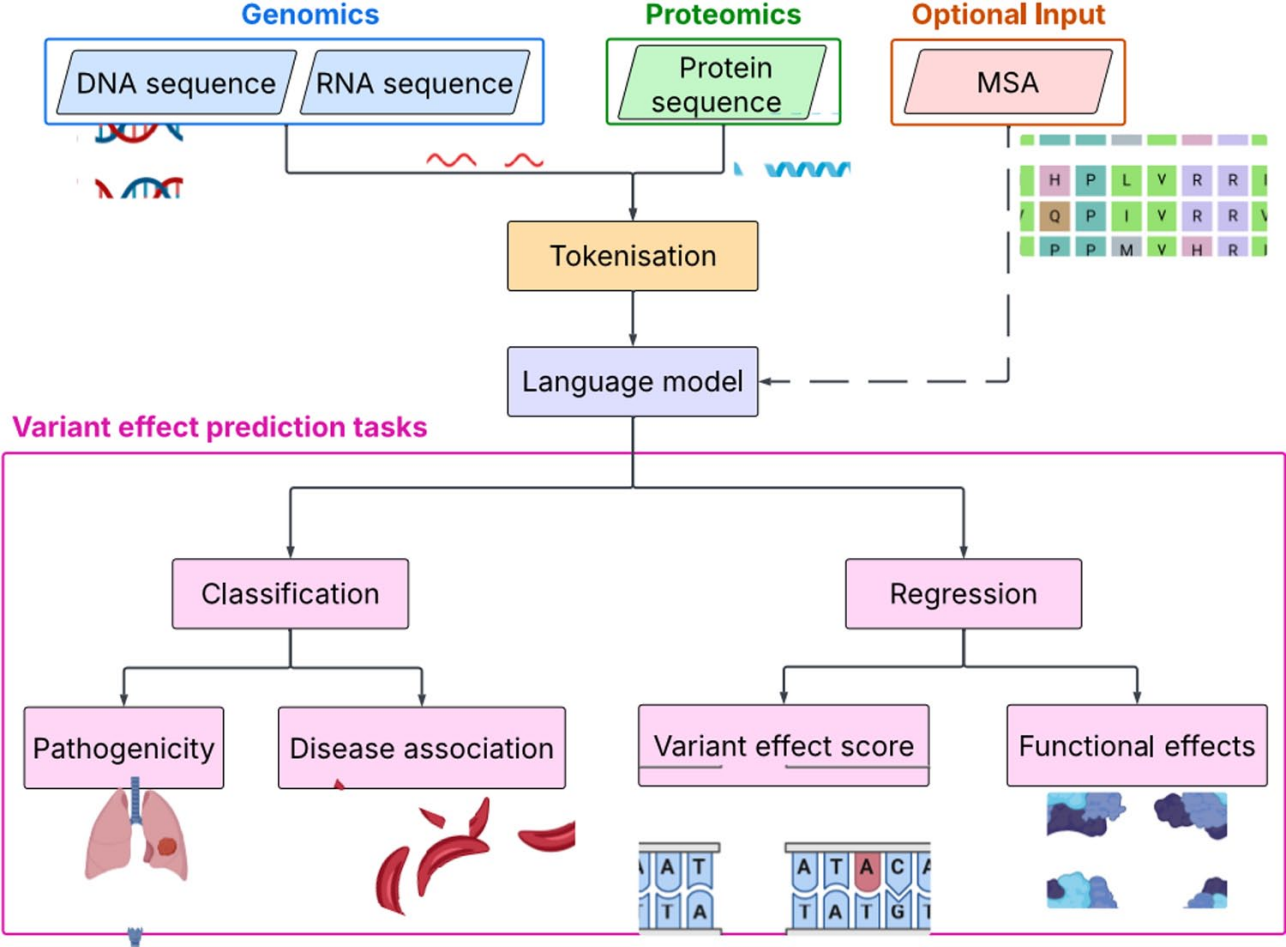
Generic language modelling pipeline, including the main categories of tasks covered in this review. The DNA, RNA, or protein sequences are tokenised before being input to the model. The model is initially pre-trained on a large corpus of data, and then fine-tuned on a data set specific to the planned downstream tasks, eg, variant pathogenicity classification. Source: Icons from BioRender https://app.biorender.com/.

Natural language processing techniques have long been used to model the structure of DNA, from statistical models^34^ to LLMs.^21^ The most frequently observed pipeline among the models reviewed here is shown in Figure 2; the sequences are tokenised before being input to the model, which is first pre-trained on a large corpus of data, and then fine-tuned for specific downstream tasks, such as the examples listed in the figure.^17,35,36^ Although unlabelled data sets of genetic sequences are abundant, labelled data sets are in shorter supply, causing a roadblock in the supervised fine-tuning of LLMs. For variant effect predictors, this can become a concern due to the lack of labelled data related to novel or emerging diseases.^37^ The field is starting to innovate to tackle this problem. For instance, a small number of models developed in recent years have circumvented the fine-tuning stage by implementing zero-shot prediction,^38^ where models progress straight from pre-training to inference, without needing additional data for fine-tuning.^39-41^ An alternative solution is data augmentation, where artificially generated examples are used to increase the size of the training data set. In genomics, this is often done by taking the reverse complement (RC) of a sequence,^42,43^ or by introducing small random translations to the sequence.^44,45^ However, a recent study presented an evolution-based method of DNA sequence augmentations, hence increasing the genetic diversity of the training data set while preserving the biological functionality of the sequences.^46^

The first step of the pipeline is tokenisation, where the input sequence is segmented into discrete units, referred to as *tokens*, using defined separators. This process converts the unstructured input data into a standardised format, hence enabling the model to create a numerical representation of the data so it can be processed.^47-49^ The chosen tokenisation method may have a significant impact on model performance. For instance, *k*-mer tokenisation produces a set of tokens with the same length *k*. The use of these constant-length tokens can lead to heterogeneous token frequencies due to the relative rarity of certain sequence patterns, such as CG dinucleotides;^50^ this can negatively impact the model training process by causing the model to focus on token frequency patterns rather than the contextual relationships within a sequence.^51^ Recent papers have addressed this limitation with the use of byte-pair encoding,^52^ which creates a frequency-balanced vocabulary by creating combined tokens for more frequent sequence patterns.^53,54^

After tokenisation, the data can be used as an input to the model. There, the selected architecture plays a key role in the quality of predictions produced; the ensuing review will analyse and compare the state-of-the-art architectures in the field. The concepts of pre-training and fine-tuning date back to the introduction of transfer learning in 1976.^55^ The pre-training stage allows the model to capture knowledge and context that can be used across a wide range of downstream tasks, while the fine-tuning stage builds task-specific understanding.^56,57^ Pre-training is most frequently done using unsupervised learning tasks such as masked language modelling (MLM), on large, unlabelled corpora of genetic sequences; this enables the model to learn without relying on the availability of large labelled data sets, which are scarce in the biomedical field.^21,58^ The smaller, labelled data sets are then used for task-specific fine-tuning. Frequently used data sets for both stages are detailed in the main review. After fine-tuning, the model can be used for downstream tasks. Figure 2 details some of the most common variant effect prediction tasks. It is important to note that there are several types of variant effect that can be measured, including fitness effect, pathogenicity, and functional change.^18^ These result in different data types, and hence, model functionality will be informed by the specific task at hand. For example, some models may aim to classify a variant as pathogenic or non-pathogenic, whereas others may look to predict a numerical value representing its functional effect.^18,59^

### Baseline clinical tools and guidelines for variant effect prediction

To understand the impact and effectiveness of the tools reviewed in this study, it is important to consider baseline clinical tools and guidelines. The simplest variant effect predictor can be built by creating a substitution matrix from a sequence alignment. For instance, the BLOSUM substitution matrix is created by aligning multiple proteins and providing a score to confer the likelihood of each amino acid substitution being conservative, ie, resulting in a protein with similar properties.^60^ In fact, these scores have proven to be somewhat effective in predicting variant pathogenicity,^61,62^ and as such provide a useful baseline to assess whether a computational approach is adding value. The first widely adopted algorithmic approach to variant effect prediction is the Sorting Intolerant From Tolerant (SIFT) algorithm,^63^ which is often used as a baseline to this day. The SIFT uses multiple alignment data to predict normalised probabilities of substitutions in each position of an input sequence. Then, a chosen cut-off is used to determine whether or not each substitution is deleterious. The original study showed that SIFT correctly predicted the deleteriousness of substitutions at a rate 14% higher than a BLOSUM62 substitution matrix; hence, it became a mainstay in the field.^63^ Another frequently used baseline tool is PROVEAN (Protein Variation Effect Analyser),^64^ an alignment-based algorithm for predicting the functional effects of variants in protein sequences, including single and multiple amino acid substitutions, insertions, and deletions. The PROVEAN achieves high results without machine learning, achieving balanced accuracy scores of over 80% for insertions and deletions and over 75% for amino acid substitutions.^64^ PolyPhen^65^ and PolyPhen-2^66^ both combine features from open-access databases to predict the effects of variants in the coding region of the human genome. The former uses empirically determined rules for classification and achieves a true positive rate of 82% on the SwissProt^67^ database. The latter uses a Naive Bayes approach and achieves true positive rates of 73% on the HumVar^68^ database and 92% on a data set compiled of Mendelian disease-causing variants and their benign counterparts from UniProt.^69^ Other widely adopted variant effect tools include the Ensembl Variant Effect Predictor^70^ and the evolutionary model of variant effect (EVE).^71^ Many of the papers reviewed below use these as baselines against which to compare their results. While there are a plethora of variant effect prediction tools and techniques, these must be held to a consistently high standard in order to be used clinically. Globally, regulators have set guidelines for reporting variants as pathogenic or benign. In the United States of America, the American College of Medical Genetics and Genomics and the Association for Molecular Pathology jointly produced a set of standards and guidelines for the interpretations of sequence variants.^72^ In the United Kingdom, the Association for Clinical Genomic Science produced a similar set of best practice guidelines, with UK-specific adaptations.^73^ These guidelines provide detailed criteria for when a variant can be reported as benign or pathogenic, based on clinical or experimental data. Although they can assist in interpreting the data when building computational variant effect predictors, these guidelines have not yet set standards in the field.

## Language Models for Variant Effect Prediction

### Pre-transformer models

Although researchers used forms of language modelling to solve machine translation as early as the 1940s,^74,75^ The Chomsky work on grammars and syntactical structures in the mid-1950s formed the basis of what we consider NLP today, where machines are able to ‘understand’ structure and context within languages.^15^ A detailed historical review of the field can be found in Sparck Jones.^76^

**Figure 3.**
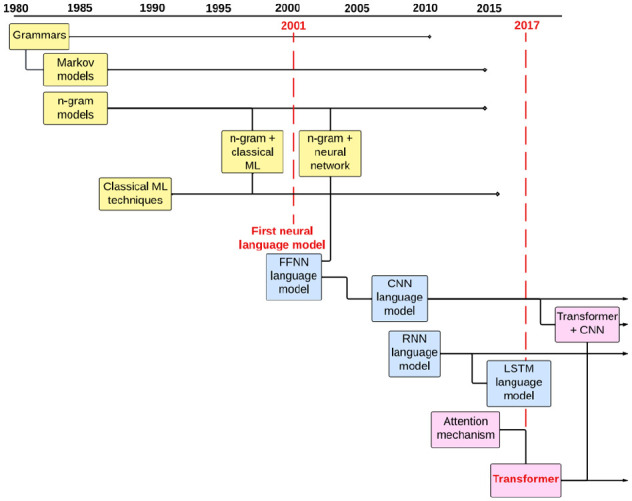
Timeline of models from 1980 until the development of the transformer. Classical ML refers to classical machine learning techniques such as support vector machine and Naive Bayes. FFNN, feed-forward neural network; CNN, convolutional neural network; LSTM, long short-term memory. Markov models are often used to construct grammars.^77,78^

Since its inception, the field has undergone many changes and innovations. Figure 3 shows the evolution of models up to the development of the transformer in 2017.

There were significant advancements in the 1980s and 1990s, with the use of statistical models such as n-gram^79^ and Hidden Markov Models.^80^ The development of neural networks led to a further turning point in the field, leading to the use of neural language models, which were better able to learn semantic relationships between words, and generalise to unseen test sets, compared to their predecessors.^81^ The original feed-forward neural network (FFNN) was created in the 1980s^82^ and adopted in language modelling in the 2000s.^83^ A widely used neural network architecture is the convolutional neural network (CNN), which was developed in the late 1990s,^84^ and introduced in NLP in the mid-2000s.^85^ Instead of relying on manually selected features, CNNs learn features directly from the input data, making them superior to implement end-to-end compared to traditional machine learning methods. Hence, they have become prevalent in DNA sequence modelling and classification.^86-89^ While CNNs are excellent at learning short-range dependencies, they struggle to model relationships between words (or nucleotides) far from each other.^90^ This limitation underscores the need for more advanced architectures to address such dependencies.

Recurrent neural networks (RNNs)^82^ were introduced in NLP as a possible alternative to CNNs, as the use of recurrent connections enabled these models to incorporate many previous inputs into future steps.^91^ However, traditional RNNs suffer from a problem referred to as ‘vanishing gradients’, which makes them prone to ‘forgetting’ inputs that are further back in the sequence. Two main alternatives have been brought forth in an attempt to circumvent this problem: (1) the long short-term memory (LSTM) network,^92^ which is able to handle long-term dependencies using a more complex architecture formed of different gates and (2) the gated recurrent unit (GRU),^93^ which uses a simplified version of the LSTM architecture to streamline sequence handling. Several variants of these models have been utilised for language modelling over the past decades, both individually and as part of ensembles with other neural networks such as CNNs.^94,95^

Despite the fact that the introduction of transformers in 2017 marked a significant milestone in deep learning, the development of models using other architectures has continued. As shown in Table 1, many recent models using pre-transformer technologies, including those using CNNs, have demonstrated notable performance. In particular, the Genomic Pre-trained Network (GPN) model, a CNN-based approach for genome-wide effects of variants in DNA, has demonstrated state-of-the-art performance.^96^ The architecture of the convolutional model was selected after it was observed that it converged faster than its transformer-based counterpart during pre-training, and the results showed that it outperformed other genome-wide variant effect predictors for *Arabidopsis*. Another noteworthy finding of this study was the performance gain observed from training on multi-species data instead of single-species data. This suggests that incorporating cross-species data can provide richer context for understanding genetic variation and can potentially improve the generalisability of the model.

**Table 1.** Summary of neural language models for variant effect prediction (see Table 5 for code/data availability).

Paper	Task	Year	Architecture	Data type	Variant type
Kim et al^88^	Identifying cancer driver mutations	2018	CNN	DNA	Coding
Pejaver et al^97^	Inferring the molecular and phenotypic impact of SAVs	2020	CNN	Protein	Coding
Shin et al^26^	Protein variant effect prediction	2021	CNN	Protein, RNA	
Dunham et al^98^	Protein variant effect prediction	2023	CNN	Protein	Coding
Benegas et al^96^	Prediction of genome-wide DNA variant effects	2023	CNN	DNA	Coding, Non-coding
Tan and Shen^99^	Non-coding variant effect prediction using genome sequence and chromatin structure	2023	CNN, GCN	DNA	Non-coding
Cheng et al^100^	Self-supervised learning for DNA sequences with circular dilated convolutional networks	2024	CNN	DNA	Non-coding

In addition to CNNs, the graph convolutional network (GCN) has also proved to be a performant non-transformer language modelling approach for variant effect prediction. Notably, its enhanced ability to capture graph-like structural information compared to other neural network architectures has proven useful in DNA variant effect prediction approaches incorporating structural data alongside sequence data.^99^

These findings underscore the ongoing relevance of pre-transformer neural network architectures in genomics and highlight the potential benefits of leveraging diverse data sets for training.

### Transformer-based models

#### History and overview

**Figure 4.**
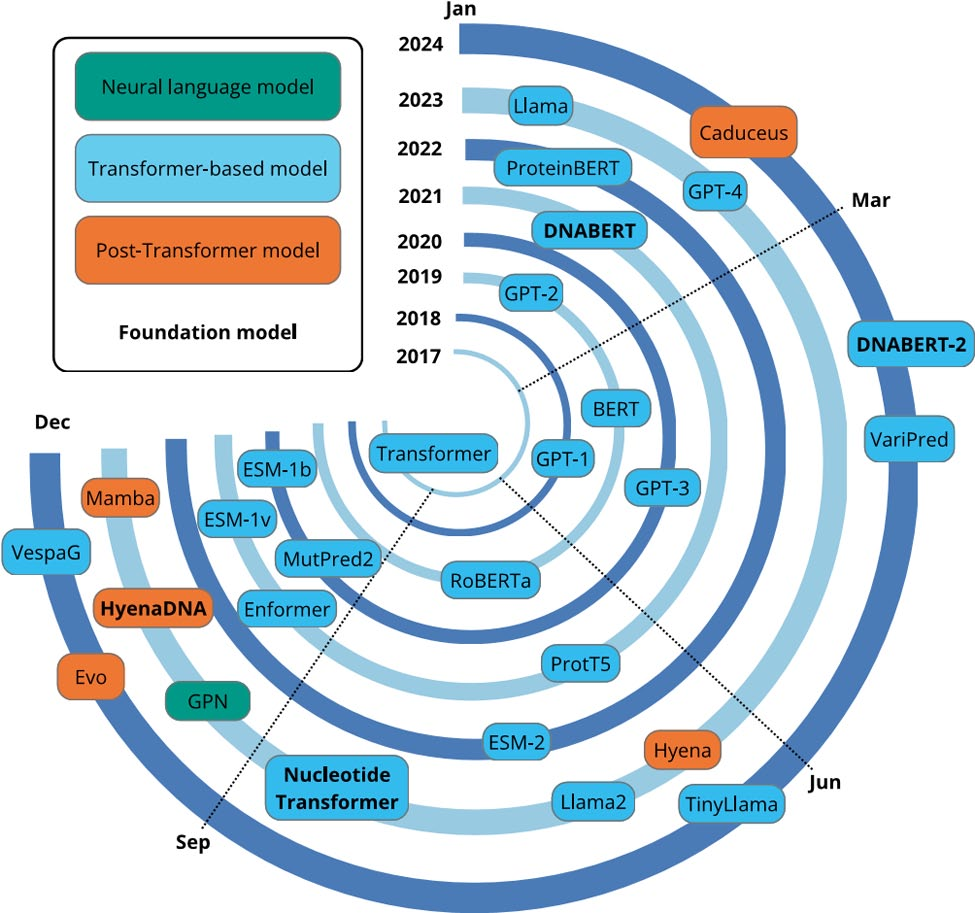
Timeline of developments in NLP since 2017.

**Figure 5.**
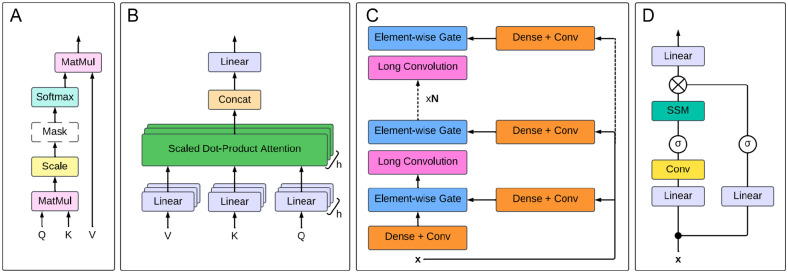
Comparison of the self-attention mechanism and alternatives. (A) Scaled dot-product attention, as shown in Avsec et al.^106^ The attention mechanism is applied simultaneously to a set of queries *Q*, with keys *K* and values *V* . Hence, the output matrix is computed as: *Attention*(*Q, K,V*) = *softmax*(*QK*^*T*^*/ *^p^[*d_k_*])*V . MatMul* = matrix multiplication. The *Mask* between the Scale and Softmax is used only in the decoder to preserve the auto-regressive property, by preventing the flow of data from right to left.^16^ (B) Multi-head attention, as shown in Avsec et al.^106^ The presence of *h* heads indicates that *h* attention layers run in parallel. (C) Hyena operator of order *N*, as shown in Nguyen et al.^107^ Combinations of dense layers and convolutions are applied to the input; the resulting projections are then fed to the element-wise gate layers. An MLP is used to implicitly parameterise the long convolutions, hence producing the convolutional filters.^107^ **x** indicates the input. (D) Mamba operator, adapted from Gu and Dao.^108^ The Mamba operator combines a state space model (SSM) with an MLP. **x** indicates the input. For the activation function *σ*, either a sigmoid linear unit^109^ or Swish^110^ is used.

**Figure 6.**
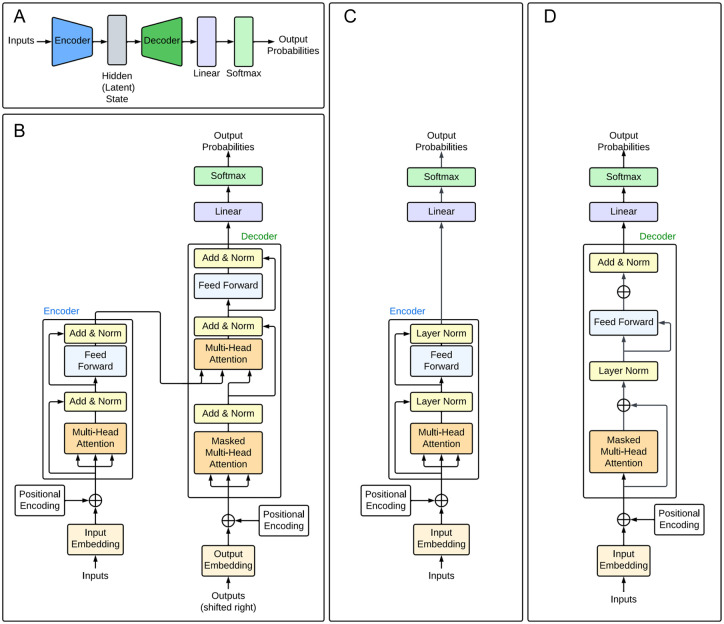
Transformer architectures. (A) High-level representation of the encoder-decoder architecture comprising the vanilla transformer architecture. The encoder encodes the input sequence into a representation, which is stored as a latent state. The decoder decodes this representation into an output sequence. This is passed into the linear and softmax layers to produce the output predictions. (B) Detailed transformer architecture, adapted from Vaswani et al.^16^ The multi-head attention modules consist of multiple self-attention modules used in parallel. These are stacked with fully connected layers to create an encoder-decoder model as shown in (A). (C) Encoder-only transformer architecture, adapted from DNABERT.^35^ (D) Decoder-only transformer architecture, adapted from GPT-1.^111^

The origination of the transformer architecture was a pivotal point in the NLP field, resulting in models that pushed the boundaries of human ability to process natural and biological languages. Figure 4 summarises the timeline of the most impactful models that have been produced, starting with the original transformer in 2017. After 7 years, it is still an active field of investigation; 2023, in particular, was a year of many developments for both transformer-based and non-transformer language models. A significant limitation common to statistical and neural language models is the need to specify a fixed context length prior to training; this restricts the capacity of these models to utilise extended contexts for predictions.^101^ The attention mechanism was created to address this limitation, by computing weights for each token in the input sequence to capture its relation to the others, and applying scaling to focus (or ‘give attention’) on the tokens relevant to the task.^102^ Several models achieved good results on machine translation tasks by combining this attention mechanism with recurrent networks.^87,103^ The attention mechanism was eventually developed into the self-attention mechanism, which forms the basis of the modern transformer.^16^ Self-attention (Figure 5) is applied within a single sequence to compute a representation of that sequence and provides a method of learning long-range dependencies within input sequences. The original transformer architecture, summarised in Figure 6A and shown in detail in Figure 6B,^16^ combines self-attention with fully connected layers. Multiple self-attention mechanisms are used in parallel; this is referred to as multi-head self-attention (Figure 5) and reduces the complexity per layer, hence increasing the capability for parallelisation. The attention layers are stacked with fully connected layers to form an encoder-decoder model. The input sequence is encoded by the encoder into a representation, which is then stored as a latent state. The decoder then decodes the representation into an output sequence, which is subsequently passed to the linear and softmax layers to produce the output predictions. The transformer architecture enables the modelling of complex patterns and long-range dependencies within sequence data, making it well-suited to tasks on DNA, RNA, and protein sequences.^22,104^ These features make the transformer more performant on complex tasks compared to recurrent or convolutional networks and enhance its efficiency. In particular, the multi-head attention mechanism enables efficient feature extraction and context-aware modelling of these biological sequences.^105^ As illustrated in Figure 3, the transformer has been used both independently and in conjunction with other models such as LSTM and CNN.

While the original transformer uses an encoder-decoder architecture, it is possible to have models consisting of only one or the other. For instance, the generative pre-trained transformer (GPT) series of models^111-113^ are decoder-only generative models, which, when given an input sequence, output the probabilities of possible subsequent tokens. By feeding the extended sequence back into the model and repeating the process many times, it is possible to generate a body of text. Figure 6D shows a decoder-only model based on GPT-1.^111^ These models have undergone significant developments since the release of GPT-1^111^ and now form the basis of the notorious ChatGPT chatbot. A significant limitation of models using the standard transformer architecture is their unidirectionality; each token can only incorporate context from the previous tokens, hence limiting the model’s ability to perform sentence-level tasks.^114,115^ This was addressed by the development of BERT (Bidirectional Encoder Representations from Transformers),^114^ an encoder-only model that transforms text embeddings into a representation that can be used for a variety of tasks. The BERT achieves bidirectionality by using a masked language modelling (MLM) pre-training objective, in which the model attempts to predict the identity of randomly masked tokens in the input sequences, hence learning a representation that combines the context from the left and right. Although originally designed to process text, BERT has also been extensively applied in the field of molecular biology, resulting in models such as DNABERT ^35^ (Figure 6C) and ProteinBERT.^116^ As the bidirectional architecture incorporates contextual information from the entire input sequence, it performs well at uncovering relationships between different elements in DNA sequences.^35^

Although LLMs have led to a paradigm shift in computational solutions for biological problems, they still experience several limitations. Data scarcity is a significant challenge; limited high-quality labelled data are available for several biological problems of interest, including non-coding variant effect prediction.^117,118^ This limits the use of LLMs for these problems due to their requirement for large quantities of training data. In addition, training on insufficiently diverse data can lead to poor generalisation across tasks.^21^ Efforts to address these limitations have led to the emergence of foundation models, LLMs which are pre-trained on very large-scale data for parameter initialisation and are then able to be fine-tuned for an extensive range of downstream applications.^119,120^ The data-intensive pre-training stage enables fine-tuning with comparatively limited data, hence improving the models’ generalisability and allowing the models to be applied to biological problems with insufficient data to train an LLM from scratch.^121^ Notable foundation models in bioinformatics, highlighted in red text in Figure 4, are DNABERT,^35^ DNABERT-2,^54^ Nucleotide Transformer,^122^ and the ESM series of models.^39,123,124^

Despite the many successes of transformers, they also have a major drawback: the time and memory used by the self-attention mechanism scale quadratically with sequence length, leading to high computational costs and creating a performance bottleneck.^125-127^ These models are hence impractical to train and use without access to extensive computational equipment and power. Crucially, this is also an environmental concern, with LLMs having huge carbon and water footprints.^128,129^ Hence, research is required to produce models that can achieve excellent results without being highly resource-intensive. These concerns have sparked a trend in the field of creating computationally efficient models as an alternative to the transformer; these are explored in detail in the next section. Notwithstanding the benefits of these post-transformer technologies, development of transformer-based models has continued, with the release of highly performant models such as DNABERT-2^54^ and VespaG^130^ as recently as 2024.

#### Review of existing models

Transformer-based LLMs are by far the most common language models used in the variant effect prediction field. This section reviews the existing models in the field, identifying key trends.

**Figure 7.**
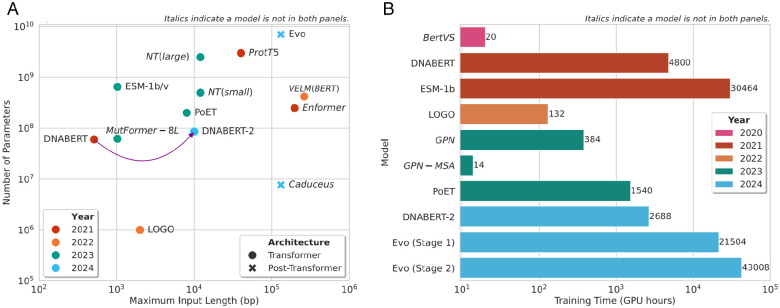
Input sequence length, number of parameters, and training time for models which have reported these statistics in the original papers. (A) Maximum input sequence length (x-axis) and number of parameters (y-axis) as reported in original papers for each model. The model names are indicated on the chart. There is no clear trend shown over time. Compared to the majority of transformer-based models, Caduceus, a Mamba-based model, has far fewer parameters and can handle longer input sequences. (B) Training time in GPU hours for state-of-the-art LLMs. GPU hours = number of hours × number of GPUs. In general, the training time required for LLMs has increased over the years. However, DNABERT and ESM-1b are outliers, having very high training times; this is likely due to the fact that both are foundation models, which were trained on very large data sets. The GPN-MSA is another outlier and has a particularly low training time, likely due to the use of retrieval augmented processing^136^ to increase computational efficiency.^43^

While all models surveyed take a sequence input – DNA, protein, or RNA – the precise input type varies. Some models take both the mutated and wild-type sequences as input,^131-134^ while others take a wild-type sequence alongside tabular data describing a variant.^97,135^ Whereas the majority of models report taking an input sequence of length up to 10 000 bases (Figure 7), the Enformer^106^ is notable as it can process significantly longer sequences, ie, up to 96 608 bases.

In addition to sequence input, several methods integrate multiple sequence alignments (MSAs) as an additional input. Indeed, the conserved residues predicted by MSA can be predictive of variant effect.^137,138^ Thus, it has been observed across many models that incorporating MSA as an auxiliary form of data improves the quality of predictions.^43,139^ However, this is largely dependent on the quality of the MSA, which is variable, and often poor due to a lack of appropriate data.^140,141^ Despite the positive results observed in variant effect predictors using MSA, they are not appropriate for all use cases, as many variants lie outside MSA coverage.^142^ In addition, several predictors not using MSA have matched or outperformed MSA-based predictors while eliminating the additional computational cost associated with having a larger training data set.^132^ For example, a benchmarking study^143^ showed that ESM-1v,^39^ which does not use MSA, outperformed several MSA-based state-of-the-art models. Hence, many recent approaches to variant effect prediction have eschewed MSA in favour of sequence-only input.

Human data are most predominantly used to train and test the models surveyed here. However, a few studies have demonstrated that incorporating data from multiple species during training can improve results compared to models trained on human data only. Indeed, it has been suggested that learning the variability across various genomes can assist a model in learning about the degree of conservation across genetic sites, hence improving its ability to predict variant pathogenicity.^43,122,144^

The majority of models surveyed adhere to the pipeline described in Figure 2, which includes pre-training and fine-tuning stages. Traditionally, language models used the pre-training task of next-token prediction. While this is still used in some contemporary models,^126^ the field has generally moved to favour MLM^114^ due to its ability to incorporate bidirectional context. However, MLM is not always the optimal choice, as it has been suggested that it may be insufficiently challenging for the model in cases where the training data include a multi-species MSA containing sequences very similar to the human genome; this has previously been addressed by excluding these very similar genomes during training.^43^

To maximise efficiency and minimise computational cost, recent work has explored zero-shot prediction, where prediction is performed straight after pre-training, without fine-tuning. A benchmarking study^17^ compared the ability of several state-of-the-art models to perform a non-coding variant effect prediction task^145^ without additional fine-tuning. There, 2 transformer models, ie, Nucleotide Transformer^122^ and Enformer,^106^ were compared with CNN models GPN^96^ and ResidualBind.^146^ Eventually, Enformer performed best, achieving a Pearson correlation of 0.68 between the experimental and predicted values. Then, the CNN methods achieved correlations between 0.35 and 0.55, whereas Nucleotide Transformer performed worst, with a correlation lower than 0.1. Based on these results, it was suggested that specialised supervised models may be a better choice for zero-shot prediction compared to current LLMs, which are pre-trained on broad data sets.^17^

While the original transformer architecture consists of an encoder-decoder framework (Figure 6A and B), the decoder portion is often not required for biological language models, as sequence generation tasks are uncommon in this field. Hence, the majority of models summarised in Table 2 employ an encoder-only framework, often based on BERT to implement bidirectionality (Figure 6C). Indeed, state-of-the-art papers have demonstrated that such architectures are able to successfully model genetic sequences without the need for a decoder.^35,39,54,123^ Still, a few encoder-decoder models, based on the original transformer,^16^ are also present.^106,147-149^ There is a lack of decoder-only models; however, this is to be expected, as such models are generally better suited to generating sequences, an ability that is not required for most variant effect prediction tasks. Furthermore, novelty does not always reside in the architecture; many models are based on pre-trained LLMs, which are then fine-tuned, hence eliminating the additional time and computational expense associated with pre-training a new model for a similar set of tasks. A prominent example is ESM-1b,^123^ which has been exploited by many studies attempting protein variant effect prediction, as shown in Table 2. Another use of pre-trained models in the field has been to provide input into models that can be considered meta-predictors.^150^ Such models input data into a pre-trained LLM, extract the output embeddings, and add a simple neural network-based classifier or regressor on top to make predictions based on these embeddings. This approach is highly data- and time-efficient in comparison to other LLM workflows, as it eliminates any training or fine-tuning of the LLM and requires only training of a simple neural network. Models using this methodology have achieved state-of-the-art results, showcasing this as an accurate and efficient framework for variant effect prediction.^106,151^ Recent work has also discovered benefits from integrating embeddings from multiple pre-trained LLMs, hence combining important context from diverse sources.^134^

**Table 2.** Summary of transformer-based language models for variant effect prediction (see Table 6 for code/data availability).

Paper	Task	Year	Architecture	Data type	Variant type
Li et al^152^	Prediction of pathogenicity of protein sequences	2020	Encoder-only (BERT)	Protein	Coding sequences
Rives et al^123^	Prediction of protein variant effects	2020	ESM-1b^123^ – encoder-only	Protein	
Meier et al^39^	Prediction of functional effects of protein mutations	2021	ESM-1v – encoder-only	Protein	
Amadeus et al^24^	Polygenic risk model for colorectal cancer	2021	Encoder-only	DNA	Coding, non-coding
Avsec et al^106^	Prediction of non-coding DNA variant effects on gene expression	2021	Encoder-decoder	DNA	Non-coding
Ji et al^35^	Identification of functional variants in non-coding DNA	2021	Encoder-only	DNA	Non-coding
Yamaguchi and Saito^153^	Prediction of variant effects on multi-domain proteins	2021	Encoder-only	Protein	
Liu et al^40^^a^	Zero-shot protein mutation pathogenicity prediction	2022	ESM-1b^123^	Protein, MSA	
Marquet et al^154^	Prediction of protein variant effects	2022	ProtBert,^155^ ESM-1b,^123^ ProtT5-XL-U50^155^	Protein	
Yang et al^156^	Prediction of deleteriousness of SNPs in non-coding DNA	2022	Encoder-only	DNA	Non-coding
Olenyi et al^157^	Predicting SAV effects	2022	Marquet et al^154^	Protein	
Zhou et al^158^^a^	Prediction of protein variant pathogenicity	2022	Elnaggar et al^155^	Protein	
Manfredi et al^135^	Prediction of SAV pathogenicity	2022	ESM-1v^39,155^	Protein	Coding
Dampier et al^159^	Prediction of protease inhibitor resistance in HIV-1 mutations	2022	Encoder-only (BERT)	RNA	
Jiang et al^160^	Prediction of SAV pathogenicity	2023	Encoder-only (BERT)	Protein	
Sun et al^161^^a^	Prediction of SAV pathogenicity from sequence and structure	2023	Encoder-only (BERT)	Protein, MSA	
Brandes et al^142^	Prediction of protein variant pathogenicity	2023	ESM-1b^123^	Protein	
Fan et al^27^	Prediction of pathogenicity of insertion and deletion variants from protein sequences	2023	ESM-1b^123,144^	Protein, MSA	
Benegas et al^43^^a^	Genome-wide variant effect prediction in human DNA	2023	Encoder-only	DNA, MSA	Coding, non-coding
Derbel et al^131^	Prediction of functional effect of SAVs	2023		Protein	Coding
Hidayat et al^147^	Prediction of BRCA1 variant pathogenicity	2023	ESM2^124^	DNA	Coding
James et al^162^	Prediction of protein-coding SAV pathogenicity in the low-density lipoprotein receptor (LDLR) protein	2023	Frazer et al,^71^ ESM-1v^39,163^	Protein, MSA	Coding
Zhou et al^54^^a^	SARS-CoV-2 variant classification	2023	Encoder-only	RNA	Non-coding
Cheng et al^164^	Proteome-wide missense variant effect prediction	2023	Encoder-only, based on Jumper et al^163^	Protein	
Danzi et al^28^	Variant prioritisation in Mendelian diseases	2023	Elnaggar et al^155^	Protein, MSA	Coding
Truong et al^148^	Prediction of protein variant fitness	2023	Encoder-decoder	Protein, MSA	
Qu et al^165^	Prediction of protein mutation effects using ensemble learning	2023	Ensemble:^16,166^	Protein, MSA	
Blaabjerg et al^139^	Protein variant effect prediction from sequence and structure	2023	Based on Rao et al^144^	Protein, MSA	
Dalla-Torre et al^122^	Prediction of DNA variant effects	2024	Encoder-only	DNA	Coding
Lin et al^132^	Prediction of protein missense variant pathogenicity	2024	ESM-1b^123^ used in twin network configuration	Protein	Coding
Wild et al^151^^a^	Prediction of DNA variant pathogenicity	2024	Benegas et al^43^ and Dalla-Torre et al^122^	DNA	Coding, non-coding
Luo et al^29^	Prediction of off-target effects of mismatches and indels	2024	Encoder-only (BERT)	DNA, RNA	
Gao et al^149^^a^	(1) Prediction of DNA variant effects (2) SARS-CoV-2 variant prioritisation	2024	Encoder-decoder	DNA, RNA	Non-coding
Zhan and Zhang^133^^a^	Prediction of coding and non-coding variant effects	2024	ESM-1b^123^	DNA, protein	Coding, non-coding
Lafita et al^167^^a^	Prediction of SAV pathogenicity	2024	ESM-1b,^123^ ESM-1v,^39^ ESM2^124^	Protein	Coding
Marquet et al^130^	Prediction of SAV effect score	2024	Shallow neural network on top of Lin et al^124^	Protein	
Yan et al^134^	Prediction of SAV pathogenicity	2024	Ensemble: ESM-1b,^123^ ESM-1v,^39^ ESM2^124,155^	Protein	Coding
Shulgina et al^168^^a^	Identification of RNA mutations beneficial to thermostability	2024	Decoder-only (GPT)	RNA	
Yang et al^169^	Pathogenicity scoring for structural variants	2024	Tabtransformer^170^	DNA	Coding
Li et al^171^	Prediction of missense coding variant pathogenicity	2024	Gated transformer	Protein	Coding
Zhong et al^172^^a^	Prediction of functional effects of protein missense variants	2024	Graph attention transformer	Protein	Coding
Linder et al^173^	Predicting the impact of genetic variation on gene expression	2025	Encoder-decoder (based on Avsec et al^106^)	DNA	Coding, non-coding
Joshi et al^174^	Prediction of coding VUS pathogenicity	2025	ESM-1b^123^	DNA	Coding
Glaser et al^175^^a^	Prediction of functional effect of protein mutations	2025	ESM2^124^	Protein	Coding

a Preprint.

By far one of the most significant transformer models has been AlphaFold,^163^ which became famous for placing first in the 13th and 14th annual Critical Assessment of Structure Prediction (CASP) competitions,^176,177^ and significantly outperforming competitors. Due to its success, this architecture was adapted for human protein variant effect prediction, resulting in AlphaMissense.^164^ This method fine-tunes AlphaFold on human and primate variant population frequency databases and classifies missense variants as likely benign, likely pathogenic, or uncertain. As in AlphaFold, an ‘Evoformer’ block^163^ is used to process encodings of residue-residue interactions and MSA. The model eventually predicts the structure of the reference sequence, and the pathogenicity score for the variant, which is then converted into a classification. The structure prediction performance of AlphaMissense is comparable to that of AlphaFold, and it performs well on variant effect prediction across a variety of data sets. AlphaMissense achieves an area under the receiver operating characteristic curve (AUROC) of 0.94 on classifying missense variants in ClinVar,^178^ outperforming EVE (AUROC = 0.911).^71^ Among a set of proteins encoded by clinically actionable genes prioritised by the American College of Medical Genetics,^179^ 77% displayed improvements in accurate pathogenicity prediction when using AlphaMissense over EVE. AlphaMissense also outperformed the state-of-the-art methods on the 2 other evaluation data sets, achieving an AUROC of >0.8 on both.

While significant developments in model architecture have occurred, work on model interpretability is still limited. The majority of models mentioned in Table 2 function as black boxes, taking an input and returning an output. Although some of them have provided promising results, it is difficult for humans to understand and interpret the underlying logic.

**Figure 8.**
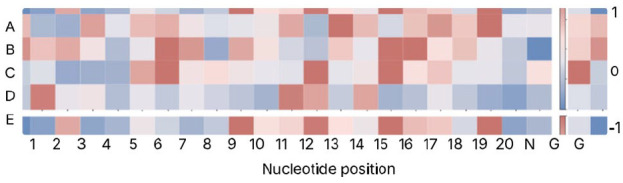
Heatmap adapted from that produced in Luo et al^29^ using the Deep SHAP method.^180^ Evaluation was done on 5 independent data sets, each for a different cell line. The y-axis denotes the data set, while the x-axis denotes the nucleotide position. The colours indicate the importance of the nucleotide position towards the predicted class label; the legend is shown on the right-hand side. 1 and −1 respectively indicate a significant positive or negative contribution. A key element of the CRISPR-Cas9 DNA editing system is the single-guide RNA sequence consisting of a 20-nucleotide protospacer and a 3-nucleotide protospacer adjacent motif (PAM) sequence.^181^ The ‘N’, ‘G’, and ‘G’ positions represent the PAM sequence, which consists of any 1 nucleotide (N) followed by 2 guanines (GG).

Currently, it is uncommon for this issue to be addressed in papers in the field; however, a recent study on predicting CRISPR/Cas9 off-target activities included interpretability as a key contribution.^29^ In the CRISPR/Cas9 gene editing system, base mismatches can occur during pairing of DNA and single-guide RNA sequences, leading to poor gene editing outcomes, and increasing the risk of ‘off-target’ mutations. Deep SHAP,^180^ a statistical method to calculate the contribution of each hidden unit to the predictions of a model, was used to evaluate the importance of specific nucleotide positions in the model’s classification of off-target or on-target for each single-guide RNA and DNA pair. This method is easily interpretable by humans and can be used to plot a heatmap to visually identify key positions that contribute significantly to the decision-making process of the model. The resultant heatmap from the paper is shown in Figure 8.^29^ The colour of each square indicates the strength of the contribution of the nucleotide position to the predicted class label; the legend is shown on the right-hand side.

The developments described above have resulted in the models described in Table 2. Comparing the performance of models across papers is challenging, as different studies tend to evaluate models on different data sets often using different metrics. One therefore cannot definitively conclude that a certain model is state-of-the-art in all aspects. It is, however, possible to assess trends across the models for specific tasks. For instance, transformer-based models have demonstrated good performance in classifying single amino acid variant (SAV) pathogenicity from protein sequences, with a number of models achieving an AUROC >0.8,^135,160,161,167^ and a few studies achieving an AUROC >0.9.^116,123,162^ The unique study published on predicting the effects of protein indels also showed promising performance; AUROC >0.8 was achieved when predicting the pathogenicity of both insertions and deletions across 2 separate data sets. However, this outperformed the previous state-of-the-art (non-transformer) methods by less than 0.1. Outcomes in predicting the functional scores of protein variants show a greater degree of variability, with the correlation between true and predicted values varying from below 0.5^139^ to above 0.9.^123^ However, this significant disparity in results may be due to the fact that these models were evaluated on different data sets. Performance on DNA variant effect prediction is similarly varied, although the best-performing models have achieved AUROC >0.9 for SNP classification.^43,133,149,156^ Although both coding and non-coding regions are addressed by these models, performance on some non-coding variant effect prediction tasks is still low; for instance, state-of-the-art models have achieved a correlation of less than 0.6 between true and predicted values on the Variant Effect Causal eQTL data set (Table 3).^106^ Existing work on RNA tasks is promising, although limited. The evaluation of 3 models on a SARS-CoV-2 variant classification task yielded a best F1-score of 73.04, indicating potential for further enhancement.^54^ Overall, the models demonstrating state-of-the-art performance across multiple tasks have been the Nucleotide Transformer,^122^ DNABERT-2,^54^ and ESM1b.^123^ These are all foundation models, the former 2 for DNA and the latter for proteins. These results suggest that foundation models represent a promising direction for future research.

**Table 3.** Most common data sets used in papers on language modelling for variant effect prediction.

Data set	Data type	Description	Size	Pub. year	No. of citations	Papers	Open access
ClinVar^182^	DNA	‘. . . germline and somatic variants of any size, type or genomic location.’^182^	500 000 variants^178^	2016	2875	Fan et al,^27^ Danzi et al,^28^ Ji et al,^35^ Liu et al,^40^, Benegas et al,^43^ Dalla-Torre et al,^122^ Lin et al,^132^ Zhan and Zhang,^133^ Yan et al,^134^ Manfredi et al,^135^ Brandes et al,^142^ Hidayat et al,^147^ Gao et al,^149^ Wild et al,^151^ Yang et al,^156^ James et al,^162^ Cheng et al,^164^ Lafita et al,^167^ and Joshi et al^174^	Yes
gnomAD^183^	DNA	Genome and exome sequences	76 215 genomes, 730 947 exomes	2020	8243	Fan et al,^27^ Danzi et al,^28^ Benegas et al,^43^ Lin et al,^132^ Brandes et al,^142^ and Jiang et al^160^	Yes
Human Gene Mutation Database (HGMD)^184^	DNA	‘. . . all known gene lesions underlying human inherited disease . . .’^184^	291 329 entries (free version) and 510 804 entries (paid version)	2020	1008	Pejaver et al,^97^ Dalla-Torre et al,^122^ Brandeset al,^142^ Yang et al,^156^ and Jiang et al^160^	Yes – free version excluding past the 3 years’ data
UniProt^185^	Protein	Protein sequences + annotations, including functional information	253 206 171 entries	2004	2900	Shin et al,^26^ Manfredi et al,^135^ Hidayat et al,^147^ and Olenyi et al^157^	Yes
CAGI5 Regulation Saturation^145^	DNA	Non-coding SNPs + effect scores	175 000 variants across 9 promoters and 5 enhancers	2019	56	Tang and Koo^17^ and Avsec et al^106^	Yes
Variant Effect Causal eQTL^106^	DNA	Non-coding SNPs + effect scores	97 563 variants^186^	2021	Unknown^a^	Avsec et al^106^ and Schiff et al^187^	Yes

Source: Data sourced from ClinVar has been employed for both training and evaluation.

ClinVar: a large open-access database of human genomic variants, is the most widely used; Pub. year: publication year; No. of citations: overall number of citations as per Google Scholar; Papers: papers in this review using the data set.

a While the paper reporting the creation of the data set^106^ has 835 citations, it was not possible to determine the number of citations for the data set itself.

**Figure 9.**
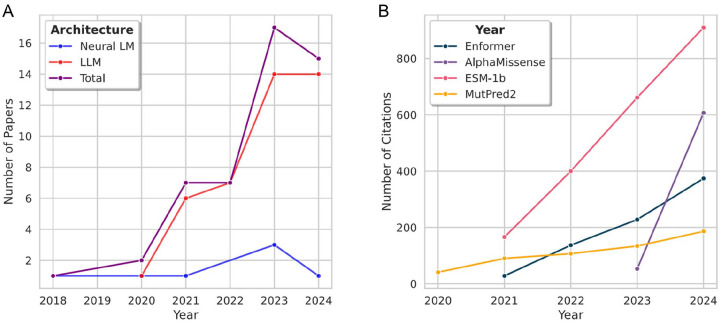
Analysis of the number of published papers and the number of annual citations for the highest-impact papers. (A) Number of papers published per year on language models for variant effect prediction, as described in Tables 1, 2, and 4. Neural LM is the neural language model (Table 1). The LLM refers to both Transformer-based and post-transformer models (Tables 2 and 4). During the period 2018 to 2024, the overall number of papers per year has generally increased, with a slight decrease from 2023 to 2024. The number of LLM papers has far exceeded the number of neural LM papers each year. (B) Number of citations per year for the most impactful papers. The number of citations per year for these papers has steadily increased since their publication.

The transformer has led to a plethora of interesting and valuable studies on variant effect prediction. However, the lack of standard evaluation data sets and protocols has made performance comparison particularly difficult. Overall, performance on protein variant pathogenicity classification has been high; however, non-coding DNA and RNA variant effect prediction tasks have proved challenging and, thus, require further investigation to improve results. Recent approaches have aimed to reduce the computational cost associated with training and testing transformer-based models alongside enhancing the prediction quality. The increasing number of papers published on such models since 2020 (Figure 9), and the fact that such papers have been published as recently as January 2025 (Table 2), suggest that the transformer remains competitive for variant effect prediction.

### Beyond the transformer

In recent years, extensions and alternatives to the self-attention mechanism have been developed in order to tackle the high computational cost currently associated with training transformer-based LLMs. The timeline of these emerging technologies is displayed in Figure 4. Figure 5 provides a visual representation of the self-attention mechanism (A), multi-head self-attention (B), and the 2 major alternatives (C and D). The first such approach to gain traction was the Hyena operator (Figure 5C), which was developed in 2023 as a direct replacement for the self-attention mechanism. Using a recurrence of multiplicative gating interactions and long convolutions,^126^ this approach scales linearly with sequence length, unlike the attention mechanism, which scales quadratically. Thus, the Hyena operator is 100 times faster than attention at a sequence length of 100 000 bases while delivering similar results.^107^ This operator forms the basis of HyenaDNA,^107^ a foundation model for DNA, which has achieved excellent results on tasks such as chromatin profile prediction and species classification. The subquadratic scaling of the Hyena operator makes it much more efficient in modelling long DNA sequences, a feature that is necessary for deciphering long-range interactions such as those involved in gene regulation.^188-190^ While current models often sacrifice single-nucleotide context to reach longer context,^35,54,106^ HyenaDNA maintains single-nucleotide resolution by using single-nucleotide tokens while also retaining the ability to process long contexts. The combination of these properties makes it well-suited to capturing the genome-wide effects of single-nucleotide alterations in DNA sequences.^107,191^

An alternative replacement for the attention mechanism is the state-space model-based Mamba operator^108^ (Figure 5D). Unlike conventional state space models, which experience performance bottlenecks due to repeated matrix multiplications, Mamba uses a structured state space sequence (S4) model, which overcomes this by employing matrix diagonalisation. During pre-training on the Hg38 human reference genome data set, Mamba scaled significantly better than the HyenaDNA and Transformer++ baselines, achieving a lower perplexity (better ability to predict the next token) with the same or fewer parameters. In addition, the Mamba perplexity continued to decrease at sequences lengths over 10^5^ base pairs (bp), whereas that of HyenaDNA increased above this length. The Mamba-based model outperformed HyenaDNA on a species classification task while using the same number of parameters, suggesting that Mamba models biological sequences more accurately and efficiently. Despite several developments in post-transformer methods, few of these models have been applied to variant effect prediction (Table 4).

**Table 4.** Summary of post-transformer large language models for variant effect prediction (see Table 7 for code/data availability).

Paper	Task	Year	Architecture	Data type	Variant type
Schiff et al^187^^[Table-fn table-fn1-11779322251358314]^	Non-coding variant effect prediction	2024	Caduceus; based on Mamba^108^	DNA	Non-coding
Nguyen et al^41^	Predicting mutational effects on bacterial protein fitness Predicting mutational effects on non-coding RNA fitness	2024	Evo, based on StripedHyena^192^	DNA, RNA, protein	Coding, Non-coding

One of the first post-transformer models applied to variant effect prediction is Caduceus,^187^ which is based on the Mamba operator.^108^ The implementation leverages the RC nature of the 2 strands in a double-helix DNA structure, recognising that both strands contain semantically equivalent information. The Mamba operator is applied twice, once to the original DNA sequence, and again to a reversed copy of the sequence; the parameters are shared between these 2 applications to increase efficiency. This double application of the operator is termed BiMamba and is used as the basis of the MambaDNA block, which additionally defines an RC mathematical operation to re-combine the forward and reverse sequences. Parameter sharing enables bidirectional models that are much deeper while using fewer parameters than transformer-based equivalents and hence are more efficient in sequence modelling. The performance of Caduceus was evaluated on a non-coding variant effect prediction data set^106^ and was compared with the state-of-the-art foundation models HyenaDNA^107^ and Nucleotide Transformer.^122^ Caduceus outperformed both state-of-the-art models, achieving an AUROC of 0.68 on variants that were 0 to 30 kbp (kilo-base pairs) from the nearest transcription start site (TSS). However, performance degraded with increasing distance of the variant from the nearest TSS, with the AUROC decreasing to 0.61 for variants at a distance of 100+ kbp. Notably, Caduceus was able to surpass the performance of Nucleotide Transformer v2 using only a fraction of the parameters (7.7M compared to the Nucleotide Transformer’s 500M).

The other notable example of a post-transformer model applied to variant effect prediction is Evo.^41^ This is a hybrid Transformer-Hyena model, where Hyena operators are combined with multi-head self-attention to improve performance on long sequences; this approach is termed StripedHyena.^192^ The majority of the computation required for sequence processing is performed by the Hyena layers, while the attention layers supplement the ability of the model to store and incorporate contextual information. Furthermore, the composition of the Hyena layers using short convolutions makes them effective at filtering ‘noisy patterns’ that can occur in DNA sequences due to the stochasticity of transcription.^41,193,194^ Evo was pre-trained on a prokaryotic whole-genome data set of 300 billion nucleotides, resulting in a model with 7 billion parameters, which can handle a context length of up to 131 072 nucleotides.^41^ Analysis during training showed that the model scaled far better with sequence length compared to state-of-the-art transformer models; while the transformer-based models scaled quadratically with sequence length, the scaling of Evo was almost linear. However, the training was highly resource-intensive, with the first stage taking 2 weeks across 64 GPUs and the second stage taking a further 2 weeks across 128 GPUs. Hence, the availability of the pre-trained model is a major contribution of this work, as it can be applied to different tasks without requiring re-training from scratch. The Evo performance on variant effect prediction was tested across 2 tasks. First, the prediction of variant effects on bacterial protein fitness. The Spearman correlation between the experimental and predicted fitness values was 0.45, underperforming compared to state-of-the-art models, including Nucleotide Transformer^122^ and RNA-FM,^195^ which achieved correlation values between 0.5 and 0.55.^41^ The second task was the prediction of variant effects on non-coding RNA fitness, in which Evo achieved a Spearman correlation of 0.27 between its predictions and the true values. While this exceeds state-of-the-art models, which achieved a correlation of less than 0.2 on the same task, the performance indicates that further research is required to produce a model that can accurately predict variant fitness in non-coding RNA. Evo was also tested on predicting mutational effects on human protein fitness; however, these experiments were unsuccessful; it was hypothesised that this may be due to the model being trained only on prokaryotic sequences, without any human samples.

These models have achieved mixed results. While in some cases, they have matched or exceeded state-of-the-art performance while reducing the number of model parameters required, the state-of-the-art models demonstrate limited ability to predict variant effects. While improvements in computational efficiency have been achieved using models such as Caduceus, this remains an area requiring further attention. For instance, Evo has achieved results exceeding the current state-of-the-art, and the pre-trained model has been made available; however, it would be necessary to undertake the resource-intensive pre-training stage again in order to make it suitable for use on the human genome. The major contribution of these post-transformer models is their enhanced ability to efficiently model longer genomic sequences, a property which is desirable for understanding long-range gene regulation mechanisms. However, the results on variant effect prediction tasks indicate that significant further work is required to use these models as reliable variant effect predictors.

## Model Evaluation

This section details the approaches to model evaluation for language models in variant effect prediction. First, the main data sets used in the field are reviewed. Then, benchmarking studies are evaluated. Finally, relevant metrics and evaluation protocols are surveyed.

### Data sets and benchmarking

A considerable challenge in the field is the difficulty of accurately comparing different models. The papers reviewed employ a variety of data sets and metrics, which seldom align. Even in the case of data sets or tasks that are used to assess multiple models, different papers select different subsets of the data set, or apply different metrics to measure model performance. This makes it challenging to compare the performance of various methods and hence can obscure the effect of different architectures on prediction quality. Hence, there is a pressing need for benchmarks that can enable comparison of models.

Table 3 summarises the main data sets used in the papers reviewed above; data sets used across multiple papers were identified, and their characteristics were summarised. The most impactful DNA database in the field is ClinVar;^182^ its coverage of many different variants across the whole human genome makes it suitable for training and evaluating a wide range of models. Two other similar databases are also very popular – gnomAD^183^ and the Human Gene Mutation Database.^184^ The former is unique due to its inclusion of several different ancestry groups from around the globe. In addition, an equivalent for proteins exists in the form of UniProt, which contains over 200 000 000 protein sequences and annotations and is exploited by several protein language models. Although these databases are used across many scientific articles, it is rare for different models to be evaluated on the same subset of a database. As shown in Tables 5 to 7, the data sets used in the field are numerous, vary significantly across papers, and frequently are not open access. Even the data sets that have been the most popular, such as the CAGI5 Regulation Saturation^145^ and Variant Effect Causal eQTL^106^ data sets, have only been employed across a small number of papers (Table 3). These constraints make it challenging to compare the performance of different models, as their data sets may vary significantly in the type of data or category of task.

**Table 5.** Code and data availability for neural language models in Table 1.

Paper	Data source	Data availability	Code availability	Model
Kim et al^88^	Hudson et al^196^ and Gonzalez-Perez et al^197^	N/A	N/A	N
Pejaver et al^97^	Stenson et al,^184^ Sherry et al^198^ and Mottaz et al^199^	http://mutpred.mutdb.org/wo_exclusive_hgmd_mp2_training_data.txt	https://github.com/vpejaver/mutpred2	N
Shin et al^26^	Apweiler et al^185^ and Riesselman et al^200^	https://zenodo. org/records/ 4606785	https://github.com/debbiemarkslab/SeqDesign	N
Dunham et al^98^	Kryshtafovych et al^176^ and AlQuraishi^201^	https://zenodo. org/records/ 7621269	N/A	https://www.ebi.ac.uk/biostudies/studies/S-BSST732
Benegas et al^96^	Sayers et al^202^ and Togninalli et al^203^	N/A	https://github.com/songlab-cal/gpn	https://huggingface.co/collections/songlab/gpn-653191edcb0270ed05ad2c3e
Tan and Shen^99^	Zhou and Troyanskaya,^204^ Chen et al,^205^ and Biggs et al^206^	https://zenodo. org/record/ 7975777	https://github.com/Shen-Lab/ncVarPred-1D3D	N
Cheng et al^100^	Avsec et al^106^ and Zhou and Troyanskaya^204^	N/A	https://github.com/wiedersehne/cdilDNA	N

**Table 6.** Code and data availability for transformer-based language models in Table 2.

Paper	Data source	Data availability	Code availability	Model
Li et al^152^	ClinVar^182^	https://www.ncbi.nlm.nih.gov/clinvar/	https://github.com/xzenglab/BertVS	Y
Rives et al^123^	Riesselman et al^200^ and Grey et al^207^	N/A	https://github.com/facebookresearch/esm	Y
Meier et al^39^	UniRef90^208^	https://www.uniprot.org/uniref	https://github.com/facebookresearch/esm	Y
Amadeus et al^24^	Yusuf et al^209^	N/A	N/A	N
Avsec et al^106^	MPRA:^145^, eQTL: Original	MPRA: http://www.genomeinterpretation.org/cagi5-regulation-saturation.html, eQTL: https://tinyurl.com/29nafrsw	https://github.com/google-deepmind/deepmind-research/tree/master/enformer	Y
Ji et al^35^	Sherry et al^198^		https://github.com/jerryji1993/DNABERT	Y
Yamaguchi and Saito^153^	N/A	N/A	https://github.com/dlnp2/evotuning_protocols_for_transformers	N
Liu et al^40^^a^	ClinVar^182,210^	https://www.ncbi.nlm.nih.gov/clinvar/	N/A	N
Marquet et al^154^	Riesselman et al^200^ and Reeb et al^211^	https://zenodo.org/records/5238537	https://github.com/Rostlab/VESPA	Y
Yang et al^156^	Rentzsch et al^212^	https://figshare.com/articles/dataset/LOGO_dbSNP_score_chr/19149827/2	https://github.com/melobio/LOGO	Y
Olenyi et al^157^	UniProt^185^ accession: Q9NZC2	https://www.uniprot.org/	https://github.com/Rostlab/LambdaPP/tree/main	N
Zhou et al^158^^a^	ClinVar^182^	https://www.ncbi.nlm.nih.gov/clinvar/	N/A	N
Manfredi et al^135^	ClinVar^182^, UniProt^185^	https://esnpsandgo.biocomp.unibo.it/datasets/	N/A	N
Dampier et al^159^	Original	https://huggingface.co/damlab	https://github.com/DamLabResources/hiv-transformers	Y
Jiang et al^160^	HGMD^184^ professional version	https://www.hgmd.cf.ac.uk/ac/index.php	https://github.com/WGLab/MutFormer	Y
Sun and Shen^161^^a^	^200^, MSA: UniRef100^208^	https://www.uniprot.org/uniref	https://github.com/Stephen2526/Structure-informed_PLM	Y
Brandeset al^142^	ClinVar^182^, HGMD^184,183^	https://github.com/ntranoslab/esm-variants	https://github.com/ntranoslab/esm-variants	Y
Fan et al^27^	Chang et al^213^ and Kaplanis et al^214^	https://github.com/xf-omics/SHINE	https://github.com/xf-omics/SHINE	Y
Benegas et al^43^^a^	ClinVar^182,183^	https://huggingface.co/collections/songlab/gpn-msa-65319280c93c85e11c803887	https://github.com/songlab-cal/gpn	Y
Derbel et al^131^	N/A	N/A	https://github.com/qgenlab/Rep2Mut	N
Hidayat et al^147^	ClinVar^182^, UniProt^185^	https://www.ncbi.nlm.nih.gov/clinvar/, https://www.uniprot.org/	N/A	N
James et al^162^	ClinVar^182^	https://www.ncbi.nlm.nih.gov/clinvar/	https://github.com/facebookresearch/esm, https://github.com/OATML/EVE	Y
Zhou et al^54^^a^	Khare et al^215^	https://github.com/MAGICS-LAB/DNABERT_2	https://github.com/MAGICS-LAB/DNABERT_2	Y
Cheng et al^164^	Notin et al,^166^ Landrum et al,^182^ Sundaram et al,^216^ Original curated Benchmark	https://github.com/OATML-Markslab/Tranception, https://github.com/google-deepmind/alphafold/tree/main/afdb	https://github.com/google-deepmind/alphamissense	N
Danzi et al^28^	ClinVar^182,183^	https://www.ncbi.nlm.nih.gov/clinvar/	https://github.com/ZuchnerLab/Maverick	Y
Truong and Bepler^148^	Notin et al^166^	https://github.com/OATML-Markslab/Tranception	https://github.com/OpenProteinAI/PoET	Y
Qu et al^165^	Notin et al^166^	https://github.com/OATML-Markslab/Tranception	N/A	N
Dalla-Torre et al^122^	ClinVar:^182^, HGMD:^184,217^	See ‘Data Availability’ section in original paper.	https://github.com/instadeepai/nucleotide-transformer	Y
Blaabjerg et al^139^	Notin et al^166^	https://zenodo.org/records/12798019	https://github.com/KULL-Centre/_2023_Blaabjerg_SSEmb	Y
Lin et al^132^	ClinVar:^182,183^	https://www.ncbi.nlm.nih.gov/clinvar/	https://github.com/wlin16/VariPred	Y
Wild et al^151^^a^	ClinVar:^182,218^	https://www.ncbi.nlm.nih.gov/clinvar/	N/A	N
Luo et al^29^	Lin et al^95^ and Chuai et al^219^	N/A	https://github.com/BrokenStringx/CRISPR-BERT	Y
Gao et al^149^^a^	ClinVar:^182,220^	See ‘Data Availability’ section in original paper.	https://github.com/ZjGaothu/EpiGePT	Y
Zhan and Zhang^133^^a^	N/A	N/A	https://github.com/zhanglab-aim/DYNA	Y
Lafita et al^167^^a^	ClinVar^182^	https://www.ncbi.nlm.nih.gov/clinvar/	N/A	N
Marquet et al^130^	Notin et al^166^	https://github.com/OATML-Markslab/Tranception	https://github.com/JSchlensok/VespaG	Y
Yan et al^134^	Manfredi et al^135^	https://github.com/yzh9607/TransEFVP/tree/master	https://github.com/yzh9607/TransEFVP/tree/master	N
Shulgina et al^168^^a^	Original	https://tinyurl.com/5abszup9	https://github.com/Doudna-lab/GARNET_DL	Y
Li et al^171^	Original	https://github.com/genemine/MVFormer	https://github.com/genemine/MVFormer	N
Zhong et al^172^^a^	Original	https://huggingface.co/gzhong/PreMode	https://github.com/ShenLab/PreMode	Y
Joshi et al^174^	UniProt^185^	https://www.uniprot.org/	Available upon request	N
Glaser and Braegelmann^175^^a^	N/A	N/A	https://github.com/moritzgls/ESM-Effect	N

a = preprint.

**Table 7.** Code and data availability for post-transformer models in Table 4.

Paper	Data source	Data availability	Code availability	Model
Schiff et al^187^	eQTL:^106^	https://tinyurl.com/29nafrsw	https://github.com/kuleshov-group/caduceus	Y
Nguyen et al^41^	Notin et al^221^ and Koboriet al^222^	N/A	https://github.com/evo-design/evo	Y

Addressing this limitation either requires the community to agree on a set of data sets on which to evaluate new models or the compilation of a framework or data set that covers several different tasks. A source of inspiration should be the CASP,^223^ a recurring set of experiments to determine the state-of-the-art in protein structure prediction methods. Every 2 years since 1994, research groups worldwide have been encouraged to submit results to ensure that a thorough and complete review of existing methods is conducted. The experiments provide a method for researchers across the community to evaluate their models on a common data set and provide several categories of tasks on which models can be assessed. This format could be highly applicable for the variant effect prediction community. A regular competition or community experiment comprising multiple categories of variant effect prediction tasks on varied context lengths would be invaluable in determining the state-of-the-art and deciding the course of future research. Furthermore, input from the clinical community on desired standards and ideal tasks could be used to assess the real-world applicability of such models.

Currently, benchmarking studies in the field are limited. The small number of existing benchmarks in the field are summarised in Table 8, with access links in Table 9. However, significant progress has been made by Livesey and Marsh at the University of Edinburgh in benchmarking protein variant effect predictors, with 2 successive studies published in 2020^140^ and 2023.^143^ They provide a comprehensive review of protein variant effect predictors at the time of publication, comparing their performance on deep mutational scanning data sets of human proteins, and ranking the models based on their results. The difference between the 2 articles highlights the progress in protein language modelling over the early 2020s. While the 2020 study identified DeepSequence^200^ – a non-language modelling, probabilistic model – as the best variant effect predictor for proteins, the 2023 one revealed that LLM methods such as ESM-1v ^39^ produced even better results. Another notable finding was the increase in data availability; in the 2023 study, there were over twice as many data sets available on which to evaluate the models. A particular strength of this study is that models were compared across multiple metrics – AUROC, AUPRC, and correlation; the benefits of this are discussed further in the metrics section. Overall, these 2 studies provide a thorough review of the existing models for protein variant effect prediction. However, language modelling of specific aspects is not explored, as deep learning models of various methodologies are assessed.

**Table 8.** Summary of existing benchmarks for large language models in variant effect prediction field (see Table 9 for access links).

Benchmark	Task	Year	Data type	No. of samples	Organisms	No. of predictors evaluated
Benchmarking of variant effect predictors using deep mutational scanning^140^	Prediction of variant effect scores for missense SAVs	2020	Protein	7239	Human, yeast, bacteria, virus	46
BEND^224^	Binary classification of non-coding SNPs as effect/no effect. (1) Gene expression and (2) disease	2023	DNA	105 263 295 495	Human	13
Updated benchmarking of variant effect predictors using deep mutational scanning^143^	Prediction of variant effect scores for missense SAVs	2023	Protein	9310	Human	55
Genome understanding evaluation^54^	Classification of SARS-CoV-2 variant pathogenicity	2024	RNA	91 669	SARS-CoV-2	
Genomics Long-Range Benchmark^186^	Prediction of SNP effect on gene expression	2024	DNAs	Avsec et al^106^: 97 563. Benegas et al^43^: 39 652. Benegas et al^43^: 2 321 473	Human	3
Benchmarking computational variant effect predictors by their ability to infer human traits^225^	Prediction of functional scores for rare-disease-associated variants in the human genome	2024	DNA	100 000	Human	24

**Table 9.** Links to the benchmarks summarised in Table 8.

Benchmark	Link
Benchmarking of variant effect predictors using deep mutational scanning^140^	https://doi.org/10.6084/m9.figshare.12369359.v1, https://doi.org/10.6084/m9.figshare.12369452.v1
BEND^224^	https://github.com/frederikkemarin/BEND
Updated benchmarking of variant effect predictors using deep mutational scanning^143^	https://figshare.com/articles/dataset/Compiled_DMS_and_VEP_predictions/21581823/1
Genome understanding evaluation^54^	https://github.com/Zhihan1996/DNABERT_2
Genomic long-range benchmark^186^	https://huggingface.co/datasets/InstaDeepAI/genomics-long-range-benchmark

Although variant-specific benchmarks are scarce, variant effect prediction tasks are included in some benchmarking studies that evaluate the performance of LLMs on genomic modelling in general. For instance, the Genome Understanding Evaluation benchmark ^54^ consists of genomic modelling tasks across multiple species, including the classification of SARS-CoV-2 variants based on sequences of 1000 bp in length. Comparison of DNABERT-2 with several different versions of DNABERT and Nucleotide Transformer showed that a version of the Nucleotide Transformer pre-trained on multi-species data performed best, while DNABERT-2 was close behind (accuracies of 73.04% and 71.21%, respectively). A complementary study is the Genomics Long-Range Benchmark,^186^ which evaluates model performance specifically on genomics tasks requiring modelling of long-range dependencies and includes the prediction of SNP effect on gene expression, using data derived from Avsec et al.^106^ It was discovered that increasing context length improved models’ ability for variant effect prediction. In addition, models with longer context lengths were able to more accurately predict the effects of variants further from the TSS. Indeed, Enformer outperformed more recent models such as Nucleotide Transformer and HyenaDNA due to its ability to handle longer context.

While past benchmarks focused on the quality of predictions, there is also a need to understand and compare the computational cost of variant effect prediction models. Recent research has highlighted the immense impact of deep learning technologies on the natural environment, from carbon emissions to water consumption.^128,129^ Transformer-based LLMs are a significant culprit due to the quadratic scaling of the attention mechanism with context length. The computational cost of training on large data sets can be extensive; as shown in Figure 7, training can span across days or weeks, using multiple GPUs. Although large foundation models such as DNABERT and ESM-1b are particularly computationally expensive to train, the training time in general has increased since 2020. However, training is not the only computational expense associated with LLMs; while training only occurs once, inference occurs repeatedly, with the frequency depending on the application of the LLM. For instance, ChatGPT was visited over 3 billion times in December 2024.^226^ Hence, since the total inference cost over time can match or exceed the training cost, it is crucial to understand and reduce its impact in the pursuit of environmentally conscious models. Table 10 lists the inference time as per the original paper for each model. Notably, not all LLM methods have high inference time, and many improve on traditional methods. In addition, recent methods have aimed to perform inference on consumer-grade machines rather than high-specification GPUs, hence making the models more accessible to run in clinical settings. For instance, VespaG^130^ took only 5.7 seconds on a 12-core CPU to make predictions for 73 unique proteins from ProteinGym,^221^ while a non-LLM method, GEMME,^227^ took 1.27 hours to perform the same task on the same hardware. However, inference time is still far less frequently reported than training time – the only models for which this is reported are listed in Table 10. Hence, it is also challenging to compare existing methods based on this criterion.

**Table 10.** Comparison of reported inference time for LLM methods.

Model	Publication year	Transformer-based	CPU/GPU	Inference time
E-SNP&GO^135^	2022	Yes	1 × 12-core CPU	12.464 seconds per variant
VariPred^132^ based on ESM-2^124^	2024	Yes	1 × GPU – 12 GB Nvidia GTX 1010Ti	0.360 seconds per variant
VespaG^130^	2024	Yes	1 × 12-core CPU	0.078 seconds per protein

Hence, to accurately compare models, simply testing on the same data set is insufficient. There must be a framework within the field for the evaluation metrics to use and the aspects of the model to report (for instance, training time, inference time, and computational resource usage).

### Metrics

#### Pre-training metrics

A significant and specific metric in NLP is *perplexity*,^228^ which can be calculated continuously throughout the pre-training stage to identify the optimal number of parameters.^41^ Language models represent sequences by calculating the probability of each token based on the context from previous tokens. The perplexity is calculated by taking the inverse probability assigned to each token in a given set of data and normalising it by the number of words, as shown in equation (1) for a data set *W* = *w*_1_*w*_2_*. . .w_N_*.^229^

(1) $$perplexity\left(W\right)\;=P{\left(w_{1}w_{2}...w_{N}\right)}^{-1/N}$$

For a given model, a lower perplexity indicates an enhanced ability to predict the next token of a sequence. However, while an improvement in perplexity often correlates with an improvement in performance on downstream tasks, this relationship is not guaranteed, and hence, further evaluation metrics are required to directly evaluate the performance of the model on the task of interest.^229,230^ For instance, although Evo achieved a lower pre-training perplexity compared to transformer-based models, the latter still achieved better Spearman correlation between true and predicted values when predicting bacterial protein fitness.^41^

#### Fine-tuning metrics

The fine-tuning metrics for NLP generally align with those for standard machine learning models. The accuracy and loss are measured throughout the fine-tuning process, which stops when the model converges (ie, when the chosen metric has remained the same for a set number of iterations).

#### Evaluation metrics

Three main categories of metrics are used to evaluate computational variant effect predictors. The first such category contains metrics that align with those used for standard machine learning models and use true and false positive rates to evaluate the predictions. These include AUROC,^43,161^ accuracy,^147,160^ precision,^159^ recall,^159^ and F1-score.^54,156^

The second category of metrics assesses the relationship between the true values and those predicted by the model. In cases where a numerical value such as a variant effect score is predicted, this is done by calculating the correlation between the 2. The Spearman rank correlation coefficient is most frequently used;^39,123,131,147^ however, some papers also use the Pearson^98,106^ correlation coefficient. All such metrics used in the reviewed papers are summarised in Table 11. While all of these metrics measure the agreement between the true and predicted values, they each measure this in a different way. For instance, the Pearson correlation coefficient assesses whether there is a linear relationship between the 2, while the Spearman correlation coefficient determines whether a monotonic relationship exists. A unique case is the Matthews correlation coefficient (MCC),^231^ which is used to evaluate the agreement between the true and predicted classes in a classification problem.^54,132^ Unlike accuracy or AUROC, it takes into account all 4 aspects of a confusion matrix (true and false positive rates, and true and false negative rates), hence better representing the overall quality of predictions produced by the model.^232^

**Table 11.** Metrics used for assessing the relationship between the values predicted by the model and the true values.

Metric	Measures	Range	Note
Pearson correlation coefficient^233^	Linear relationship	(Strong negative) −1 to 1 (strong positive)	0 = no relationship
Spearman correlation coefficient^234^	Monotonic relationship	(Strong negative) −1 to 1 (strong positive)	0 = no relationship
Matthews correlation coefficient^231^	Agreement of classes	(All inverse) −1 to 1 (all correct)	0 = no agreement
Jaccard similarity index^235^	Similarity	(No similarity) 0 to 1 (complete similarity)	

Pearson, Spearman, and Jaccard metrics are used for prediction of numerical values. Matthews is used for classification.

To compare the agreement of these 2 categories of metrics, a simple meta-predictor was created by using the pre-trained Enformer model^106^ to generate embeddings from SNPs in the ncVarDB^206^ and using a simple machine learning classifier on top to perform a binary pathogenicity classification. The results of the different models tested are displayed in Table 12. It must be noted that, while Random Forest and Gradient Boosting achieved the same accuracy, their AUROC and MCC were different. In addition, the MCC achieved using support vector machine (SVM) with a linear kernel is very similar to that achieved using Random Forest, despite the latter having higher accuracy and AUROC values. These results demonstrate the importance of evaluating and comparing models across these different dimensions in order to fully understand the differences and determine the state-of-the-art.

**Table 12.** Comparison of metrics for models performing variant pathogenicity classification on SNPs from ncVarDB,^206^ using embeddings extracted from Enformer.^106^.

Model	Accuracy	AUROC	MCC
SVM (RBF kernel)	69.4%	0.725	0.516
SVM (linear kernel)	73.6%	0.763	0.574
Random forest	77.5%	0.781	0.572
Gradient boosting	77.5%	0.778	0.558

MCC: Matthews correlation coefficient.

Beyond perplexity, no further NLP-specific metrics have been used to evaluate variant effect predictors based on language models. However, many such metrics have been developed to evaluate the ability to model natural languages, such as ROUGE^236^ and its variants, and a variety of semantic embedding-based metrics.^237,238^ Moreover, recent papers have investigated the use of semantic similarity for assessing the ability of LLMs to appropriately model natural languages. Of particular interest is a 2024 paper in which the ability of an encoder to model substitution of a word with a synonym or antonym is tested;^239^ this concept could be extended to genetic language modelling and hence evaluate the ability of an encoder to model substitution of a nucleotide. Despite the ability of non-NLP-specific metrics to evaluate the results of a model, they have no ability to assess the quality of language modelling or understand the underlying logic. Hence, to fully understand LLM performance, standard metrics must be combined with NLP-specific metrics.

While there are several metrics to assess the quality of model predictions, looking solely at the values of these metrics does not take into account other key aspects of a model, including computational cost. Although modifications such as including additional features in the training data, or increasing the size of the model, can enhance the predictive performance, they can also lead to a significantly higher computational cost. This calls into question the extent to which an increase in computational cost is justified for a corresponding increase in prediction quality.^240^ For instance, usage of Pareto optimality has been adopted to attempt to select models with an appropriate trade-off between accuracy and inference latency.^241^ In the future, it would be very valuable to define a metric to combine the information from each of the 3 categories above with data regarding computational cost.

## Discussion

The advent of the transformer model in 2017 led to a paradigm shift in NLP and its applications to various fields, including the prediction of biological variant effects. Transformer-based language models have achieved mixed results in this area; while some models excel, others fail to make accurate predictions. Another significant limitation of transformers is the overwhelming computational cost associated with training and inference due to the quadratic scaling of the cost of the attention mechanism with sequence length. Research to address this has led to the development of several attention alternatives such as Mamba and Hyena. While these have garnered much attention in the LLM field, their capacity for variant effect prediction has not yet been fully explored, with only 2 models being used for this application so far. In addition, transformer-based models are still being proposed for variant effect prediction, as recently as early 2025,^174^ demonstrating that this technology remains competitive.

The models produced to date have focused largely on single-nucleotide substitutions within proteins, or protein-coding regions of the human genome, often achieving promising results. However, there has been very little work on multiple base-pair variants, or non-substitution variant types, such as indels. Furthermore, while there has been extensive work on modelling DNA and protein sequences, there has been limited work on human RNA, despite the known associations between RNA variants and disease.^242,243^ Moreover, although extensive research has been conducted on the effects of variation within the human genome, very few recent studies have investigated the effects of variants in pathogenic organisms and viruses with a high disease burden. In particular, only 2 studies^54,149^ have looked at the mutational effects of SARS-CoV-2, which had a devastating impact on human health during the COVID-19 pandemic. Still, some work has been conducted on using deep learning to viral mutation data to predict individual risk^244^ and the possibility of drug resistance.^245^ Moreover, given that LLMs have already demonstrated effectiveness in modelling HIV,^159^ they could potentially enhance results in this area.

Despite significant advancements in recent years, the field still faces several limitations. Many of the most prominent challenges are related to data rather than model architectures. A common issue observed among computational variant effect predictors is *type 2 data circularity*. Studies found that, in many cases, all variants within a particular gene are recorded with the same label (pathogenic or benign) across multiple different variant databases. This leads to models trained on these data performing well on known variants in known genes but poorly on de novo variants for newly identified risk genes.^246^ Although a benchmarking study investigating this issue found that traditional machine learning models were the most prone to suffering from this issue, only 1 LLM (an ESM variant) was tested; hence, it is possible that others may still be at risk of suffering from this issue.^132^ Therefore, it may be of interest to include such a test in future LLM benchmarking studies.

Another significant data-related challenge is that of demographic bias. Many large genomics data sets, such as UK Biobank, contain data largely from individuals of White European descent.^247^ This poses a concern, as several mutations related to Mendelian diseases, including sickle cell anaemia and Tay-Sachs disease, have been shown to differ significantly in prominence across different groups.^248,249^ Hence, training on an ancestrally homogeneous data set risks the loss of valuable features when modelling the human genome and can lead to poor generalisation of models across different ancestral groups. The computational health care field has largely continued to uphold existing biases against underserved groups, with some widely used algorithms displaying clear racial bias.^250^ As the field moves forward into an era where algorithms play an increasingly pivotal role in shaping personalised medicine, it is crucial to prioritise equity in future developments to ensure fair and unbiased outcomes for all.

In addition to addressing data set composition, the privacy of patient data is another key consideration when using LLMs for health care-related applications. As LLMs have already demonstrated their ability to identify sensitive information in documents such as electronic health records,^251,252^ this raises concerns around accidental patient identification via training data. Genomic data must be treated as particularly sensitive, due to the possibility of identifying not only an individual but also their familial relationships and links to specific traits or diseases.^253^ This is of particular concern in rare disease research, where access to data on diseases experienced by only a handful of individuals increases the risk of individuals being identified. Although privacy solutions for genomic data sharing are being rapidly explored and developed,^254,255^ it is crucial to consider these through the lens of LLMs and the handling of data by those who develop these models. Indeed, LLMs can be susceptible to Membership Inference Attacks (MIAs)^256^ and User Inference Attacks (UIAs).^257^ The MIA aims to determine whether a given data record is present in the training data of an LLM and is conducted by creating an adversarial model to recognise the differences in an LLM’s response to its training data and its response to other samples. Recent research has shown that such attacks are effective on clinical language models, with samples from individuals with rare diseases being at greater risk of privacy leakage.^258^ On the contrary, UIA attempts to ascertain whether an individual’s data were used in fine-tuning an LLM. While MIA threatens the privacy of individual samples, UIA puts the privacy of users who have contributed multiple samples at risk.^257^ Both sets of attacks can severely compromise patient data privacy and can lead to the revelation of sensitive information about participants. However, tests on MIA and UIA have not yet been applied to genomic language models, and the latter has not yet been tested for any clinical LLMs. Hence, a framework must be created for testing the resiliency of state-of-the-art models in the field against such attacks. Crucially, these tests must be performed before models are adopted into clinical settings, to avoid putting patients at risk. The lack of interpretability in existing models poses another significant concern. The LLMs, like many deep learning models, often function as ‘black boxes’, with little human-understandable logic connecting the input data to the decisions of the model. In order to trust the decision-making of such models in clinical settings, it is necessary to understand and validate the logic behind such decisions. Due to the limited interpretability of existing predictors, meta-predictors based on these models experience the same issues. For instance, some studies have proposed that the exclusive use of LLM embeddings leads to limited biological interpretability and suggested that including more protein structure data could make the model more interpretable without increasing training costs.^134^ Although work on interpretability in the field is currently limited, 1 recent paper^29^ explores a method based on SHAP^180^ to calculate the relative importance of the model’s hidden features on the output score. The authors reported this method to be effective, and it translated well to a visual representation of the relationship between the input and output features (Figure 7). However, this approach has yet to be adopted across other variant effect prediction papers. Alternatively, in attention-based models such as transformers, the attention weights confer the relative importance given to certain tokens^259,260^ and hence can be used to infer the impact of certain tokens in the decision of the model.^261^ While this approach has been used to interpret the results of transformer-based for a number of bioinformatics applications,^262-264^ it has not yet appeared in the literature for variant effect prediction. For high-dimensional models, t-distributed Stochastic Neighbour Embedding (t-SNE)^265^ is able to visualise the relationships between features and predictions. Thus, it has been used in transformer-based protein language models.^266^ While some excellent reviews have been published on the state of interpretability of LLMs in biology and medicine,^267^ they focus only on attention-based models. Further research is required to incorporate interpretability into post-transformer models such as Mamba and Hyena.

### Future trends

Due to the significant training and inference costs associated with transformer-based LLMs, many recent studies have focused on creating more computationally efficient models, either using transformers or substituting the attention mechanism with alternative operators such as Hyena or Mamba. Although the advent of small language models (SLMs)^244^ has advanced this area of research in natural language-based LLMs, they have not yet been applied to genetic sequences. A notable SLM is TinyLlama,^268^ which utilises the same architecture and tokeniser as Llama2,^269^ while leveraging novel computational methods such as FlashAttention^270^ to create a model with fewer parameters and increased computational efficiency compared to state-of-the-art LLMs. The SLMs have already demonstrated impressive performance in text classification^271^ and text-based health monitoring,^272^ matching or exceeding the results achieved using LLMs. These findings underscore the potential of SLMs in future research and suggest that they may be an interesting avenue of advancement for biological language modelling also.

Although the development of SLMs is on the horizon, LLMs continue to be widely used. Recent papers have shown a trend towards the use of foundation models, which are pre-trained on a large corpus of data and can be fine-tuned for a wide range of downstream tasks. For instance, 8 separate papers in Table 2 base their models on the ESM-1b^123^ foundation model. As the field aims to reduce computational cost, it is likely that foundation models will be even more widely used as an alternative to ab initio pre-training of new LLMs.

As the number of models in the field rapidly increases,^5^ often trained and evaluated on different data sets, it is becoming increasingly challenging to identify the true state-of-the-art. To address this rising need, the development of benchmarking data sets has accelerated since 2023, resulting in the creation of benchmarks such as the Genome Understanding Evaluation.^54^ As interest in computational efficiency and model fairness grows, it is likely that future benchmarks will include methods to assess these features of models and that such measures will become more significant when comparing models. Moreover, although models may perform well during technical evaluations, it is crucial to define and adhere to specific standards in order to discern their efficacy in clinical settings. For instance, in 2018, the National Health Service (NHS) in England and the UK National Institute for Health and Care Excellence (NICE) developed an evidence standards framework^273^ to provide guidance on the development and usage of digital health and care technologies. While this framework places a high emphasis on demonstrating valuable results and significant benefits to the target population, it is not specific to artificial intelligence (AI) or LLM-based technologies, and hence does not detail any expectations for numerical results or other aspects of models. It is therefore of the utmost importance that those in the computational field work closely with clinicians to decide appropriate standards for the performance of variant effect predictors and implement strategies to bridge the gap between research and practice. Existing frameworks for models predicting individual prognosis or diagnosis include TRIPOD,^274^ which explores transparent reporting, and PROBAST,^275^ which estimates the risk of bias – these could be used to inform the creation of similar frameworks for language model-based variant effect predictors.

Alongside appropriate performance, the adoption of computational models in the clinical field requires the exploration of clinically relevant problems. While the bulk of work in the field has focused on the coding regions of the genome, research continues to uncover associations between non-coding variants and rare but highly impactful diseases in humans.^276-278^ Thus, although there has recently been increasing interest in predicting the impact of human genetic variation in the non-coding regions, further computational exploration of the non-coding genome is required. Furthermore, although current research focuses mainly on SNPs, diseases such as haemophilia have been linked to multiple base-pair variants or combinations of co-occurring SNPs.^279,280^ Very few papers exist on computational prediction of the effects of such variants;^281,282^ hence, this is an area of great interest for future work.

## Conclusion

Although language models have proven effective in modelling DNA, RNA, and protein sequences, their results on variant effect prediction tasks remain mixed. The best performance on these tasks has been achieved by large transformer-based foundation models, pre-trained on large corpora of sequence data. However, such models incur a high-computational cost in terms of training and inference. While this has begun to be addressed via the creation of alternatives and extensions to the attention mechanism, these have had limited use in bioinformatics thus far. Initial studies show that models based on these technologies, such as Caduceus and Evo, achieve results comparable to transformer-based models while consuming less time and fewer resources for training and inference. Nevertheless, the state-of-the-art results for some tasks of importance, including non-coding variant effect prediction, require improvement. Despite the substantial progress in the field in recent years, there are still a number of limitations that persist, including demographic bias in training data sets, and the limited work on variants spanning multiple base pairs or situated in the non-coding regions of the genome.
